# A reduced-toxicity myeloablative conditioning approach for hematopoietic cell transplant in juvenile myelomonocytic leukemia

**DOI:** 10.3389/fonc.2025.1541192

**Published:** 2025-06-03

**Authors:** Eman Elsabagh, Rachel Gallant, Lior Goldberg, Aditya Sharma, Paul L. Martin, Timothy A. Driscoll, Andrea Bauchat, Joanne Kurtzberg, LaTarsha Spencer, Paibel I. Aguayo-Hiraldo, Neena Kapoor, Kris M. Mahadeo, Hisham Abdel-Azim

**Affiliations:** ^1^ Department of Pediatrics, Division of Transplantation and Cellular Therapy, Duke University, Durham, NC, United States; ^2^ Division of Pediatric Hematology-Oncology, University of Oklahoma Health Sciences Center, Oklahoma City, OK, United States; ^3^ Department of Pediatrics, T Cell Therapeutics Research Laboratories, City of Hope National Medical Center and Beckman Research Institute, Duarte, CA, United States; ^4^ Division of Transplant/Cell Therapy and Hematological Malignancies, Cancer Center, Departments of Pediatrics and Medicine, Loma Linda University School of Medicine, Children Hospital and Medical Center, Loma Linda, CA, United States; ^5^ Section of Blood & Marrow Transplantation, Cancer and Blood Disease Institute, Children’s Hospital Los Angeles, Los Angeles, CA, United States

**Keywords:** JMML, pediatric, conditioning regimen, hematopoietic stem cell transplant, busulfan, melphalan

## Abstract

**Introduction:**

Allogeneic hematopoietic cell transplantation (HCT) is a potentially curative treatment for most children with juvenile myelomonocytic leukemia (JMML), but overall survival remains poor at 50%. Given its rarity and heterogeneity, there is no standard HCT conditioning regimen for JMML.

**Methods:**

Retrospective study of consecutive patients with JMML who underwent HCT using a busulfan/ melphalan backbone conditioning regimen (n=17) at two academic centers.

**Results:**

The median age at HCT was 1.9 (range 0.7-6.0) years. At a median follow up of 7.6 (range 2.9-21.5) years, 100% disease-free (DFS) and overall survival (OS), with prompt immune reconstitution were observed. This cyclophosphamide-sparing approach was associated with no transplant related mortality.

**Discussion:**

Given excellent clinical outcomes at extended follow-up, prospective studies are needed to confirm our findings in this ultra-rare disease.

## Introduction

There are approximately 25 cases/year of juvenile myelomonocytic leukemia (JMML) diagnosed in the United States, an aggressive myeloproliferative/myelodysplastic disorder characterized by infiltration of peripheral blood, bone marrow, and organs by abnormal myelomonocytic cells (1). Primarily a disease of infancy/childhood, the majority have somatic and/or germline mutations within the RAS/MAPK signaling pathway (*NF1*, *PTPN11, KRAS, NRAS*, or *CBL)*, leading to pathologic activation and hypersensitivity of myeloid progenitor cells to the granulocyte-monocyte colony stimulating factor (GM-CSF) (1, 2). Prior to molecular characterization, *in vitro* hypersensitivity of monocyte/macrophage colonies to GM-CSF represented a diagnostic tool. Untreated, the median survival is less than 12 months (3). Neurofibromatosis type 1 (NF-1) mutations, found in up to 30% of JMML cases, significantly influence prognosis/treatment. This underscores the complexity of JMML and necessity for tailored therapeutic strategies (4). While affected children with germline *CBL* mutations may have spontaneous regression of myeloproliferation despite the persistence of LOH of *CBL* in hematopoietic cells, allogenic hematopoietic stem cell transplant (HCT) is recommended with disease progression (5).

HCT is the only potentially curative therapy for children with JMML. Yet, despite intensive therapy, 5-year overall survival (OS) following HCT is poor (50%); the primary cause of mortality following HCT, is relapse at a median of 4 months (6). Chemotherapy alone may provide temporary remission but is not curative (6, 7). Given the rarity and heterogeneity of JMML, no standard HCT approach has been established.

Total body irradiation (TBI) has been replaced by chemotherapy-based conditioning due to associated toxicity in younger children (6–8). Favorable outcomes with a TBI-sparing approach to JMML have been previously reported (8). This report characterizes long-term outcomes associated with that approach in an expanded cohort.

## Material and methods

**Figure 1:**
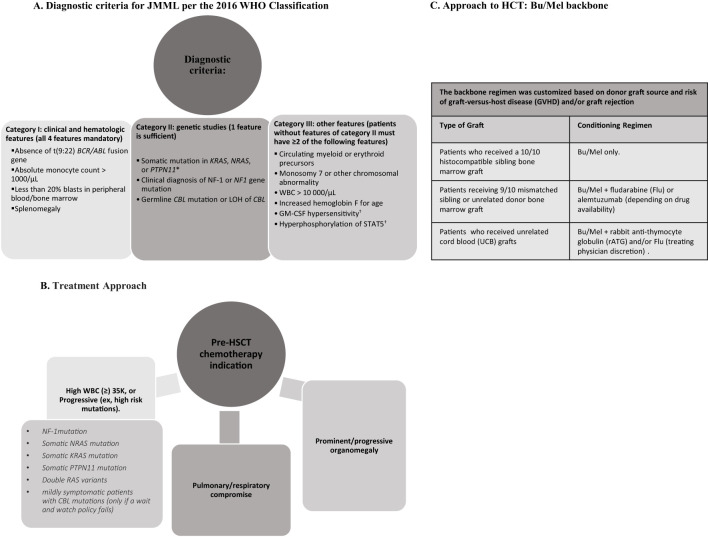
A comprehensive infographic summarizing diagnostic criteria, pre-treatment considerations, treatment approach for hematopoietic cell transplantation (HCT). **(A)** Diagnostic criteria for JMML based on clinical and hematologic features, genetic studies, and other supplementary features. **(B)** Pre-HCT chemotherapy indications in JMML. **(C)** Backbone regimen customized by donor graft source.

This retrospective study (approved by respective institutional review boards) includes all patients diagnosed with JMML undergoing first HCT between 2002-2022 at Children’s Hospital Los Angeles (CHLA) and Duke Children’s Hospital (Duke). Approach to diagnosis and indications for HCT are shown in **Figure 1**. Patients received myeloablative conditioning (MAC) with a busulfan/melphalan (Bu/Mel) backbone; Bu 1 mg/kg every 6 hours intravenously (IV) on days -8 to -5 (with therapeutic drug monitoring [TDM] targeting overall concentration steady state [CSS] of 800-1000 ng/mL) and Mel 45mg/m^2^/day IV on days -4 to -2. The backbone regimen was customized based on donor graft source and risk of graft-versus-host disease (GVHD) and/or graft rejection (**Figure 1**). Sinusoidal obstructive syndrome (SOS) was retrospectively graded (9). All patients either received intravenous immunoglobulin (IVIG) (1gm/kg) (CHLA) 48 hours prior to graft infusion to block the reticuloendothelial system (10) or underwent pre-HCT splenectomy (Duke) (6). Intended time for initiation of immunosuppression withdrawal was D+100, if no evidence GVHD.

## Results

Median age at diagnosis (n=17) was 0.9 (range 0.4-4.1) and age at HCT was 1.9 (range 0.7-6.0) years, respectively. Median time from diagnosis to HCT was 6 months with 76.5% patients receiving chemotherapy prior to HCT. Though underlying molecular lesions were not identified(n=8) in all patients due to the retrospective nature of the study and limitations of the assays available then, this cohort includes patients with high-risk molecular features such as *NF1*, *KRAS*, and *PTPN11* mutations. All patients had clinical and morphological bone marrow progression of their disease, prior to HCT, irrespective of the molecular features (**Table 1**). Median (range) Bu CSS and area under the curve were 884 (560-1096) mg/L and 1293 (819-1601) mmol/L-minute, respectively. Median total nucleated cell counts and CD34 cell dose were 10 × 10^8 cells/kg (5-15 x10^8) and 9.5 × 10^6 cells/kg (2.2-15 x10^6), respectively. Median time to neutrophil and platelet engraftment were 17 (range: 12-43) and 52 (range: 15-133) days, respectively. No patients developed vasculitis pre- or post-HCT and no transplant related mortality was observed. Five (29%) patients developed SOS [Mild (n=4) and severe (n=1)] which resolved without specific therapy. Infectious complications [sepsis (n=4) and cytomegalovirus (CMV) reactivation (n=2)], resolved with therapy. Indications for PICU admission included severe SOS (n=1), respiratory distress (n=1), pericardial effusion (n=1), and sepsis (n=2). One patient developed grade IV acute GVHD which progressed to chronic GVHD requiring bowel resection and systemic therapy. At 13.9 years follow-up, this patient is alive with a performance score of 100%.

**Table 1:** Clinical characteristics and outcomes for pediatric patients with juvenile myelomonocytic leukemia (JMML) treated with hematopoietic cell transplant (HSCT) using busulfan/melphalan conditioning regimen.

	Demographics			At Diagnosis						HCT			HCT Complications and Outcomes					
Pt	Age at dx (yrs)	Age at HCT (yrs)	Sex	WBC	Plt	BM blast (%)	Hb F (%)	Mutation	Chemo	Conditioning	Graft Type/	TNC/kg x108	Graft failure	GVHD prophylaxis	GVHD	GVHD Treatment	Chimerism	Alive Follow-up Time (yrs)
1	0.6	0.8	F	60.00	89.0	8	18.0	Extra small ring ch(47XX, +r(3\E\46XX).	Flu/Ara-C, RA 6MP	Bu/Mel/rATG	4/6 UCB	2.25	N	Tacrolimus, MPS	Y Acute Gr 2 Skin	MPS	100%	Yes 15.2
2	4	4.2	F	30.4	37.0	9	52.0	None	None	Bu/Mel/Flu	9/10 MMSD	4.2	N	Tacrolimus, MPS	Y Acute Gr 4 Skin, Liver, Gut Chronic Gut	MPS,Dacluzimab bowel resection	100%	Yes 13.9
3	0.5	1.2	M	36.3	44.0	6	7.7	None	6MP	Bu/Mel/rATG	5/6 UCB	0.7	N	Tacrolimus, MPS	N	NA	96%	Yes 10.5
4	1.7	2.5	F	25.3	110.0	3	0.5	None	6MP	Bu/Mel	10/10 MSD	4.5	N	Tacrolimus, MTX	N	NA	99%	Yes 9.2
5	0.8	3.8	M	130.00	43.0	5	4.8	None	HU, 6MP	Bu/Mel	10/10 MSD	11.3	N	Tacrolimus, MTX	Y Acute Gr 3 Skin	MPS	100%	Yes 7.8
6	1.4	2.2	F	20.00	19.0	3	7.6	TPN11 11.182A>T	None	Bu/Mel/rATG	6/6 UCB	0.5	Y	Tacrolimus, MPS	N	NA	6% (1st BMT)* 100%(2nd BMT)	Yes 7.7
7	2.5	3.2	M	38.90	22.0	3	42.2	PTPN11 11.182A>T	6MP	Bu/Mel/Alem	9/10 MUD	2.7	N	Tacrolimus, MTX	Y Acute Gr 1-2 Skin Gr 2 Gut	MPS, topical steroids, Azathioprine, Basiliximab, ECP	100%	Yes 7.6
8	0.5	0.9	M	125	9	7	3.2	KRAS c.35G>T p. Gly2Val	Flu/Ara-C, 6MP	Bu/Mel/Alem	10/10 MUD	2.5	N	Tacrolimus, MTX	N	NA	100%	Yes 6.9
9	0.42	2.0	M	25	237	0	4.6	None	FLAG-Ida	Bu/Mel/Alem	10/10 MUD	2.3	N	Tacrolimus, MTX	Y Acute Gr 1 Skin	Topical steroids	100%	Yes 6.8
10	3.3	3.6	M	25.72	35	7	NR	None	None	Bu/Mel/Flu	10/10 MUD	4.9	N	Tacrolimus, MTX	Y Acute Gr 1 Skin Gr 2 Gut Chronic Skin (Ltd)*	Topical steroids, MPS, Tacrolimus	100%	Yes 6.3
11	0.4	0.7	F	258.8	243	7	NR	NF1 c.7178delA (germline) c.1105C>T (somatic)	Flu/Ara-C+ ADE	Bu/Mel/Alem	9/10 MUD	10.0	N	Tacrolimus, MTX	N	NA	96.72%	Yes 4.3
12	4.11	6.0	M	33.7	75.0	0	1.2	NF1 c.1019_1020del TET2 c.4062_4063del. ASXL1 c.1919dup c.4004del	Flu/Ara-C	Bu/Mel/Alem	9/10 MUD	0.7	N	Tacrolimus, MTX	N	NA	100%	Yes 3.0
13	0.9	1.4	M	23	191	5	NR	None	Flu/Ara-C, RA	Bu/Mel/Flu/ATG	6/6 UCB	0.6	N	CSA, MMF	Y Chronic Skin-		100%	Yes 6.5
14	0.6	0.9	M	23.9	123	4	14.4	PTPN11, c.226G>A (somatic)	Flu/Ara-C	Bu/Mel/Flu	5/6 UCB	0.99	N	CSA, MMF	Y Chronic Skin-	Steroid, Ruxolitinib	100%	Yes 2.9
15	0.5	0.9	M	38.9	383	4	NR	NF-1(germline)	None	Bu/Mel/Flu/ATG	5/6 UCB	2.7	Y	CSA, MPS	N	NA	<10%*	Yes 18
16	0.9	1.9	F	29.5	163	5	22	None	Flu/Ara-C, RA	Bu/Mel/ATG	5/6 UCB	1.4	N	CSA/FK, MPS	N	NA	90%	Yes 21.5
17	1	1.4	M	14.6	57	NR	1	KRAS (c.38G>A, p. Gly13Asp (somatic)	Aza	Bu/Mel/Flu	5/6 UCB	1.7	N	CSA, MMF	N	NA	100%	Yes 6.8

Pt, patient, dx, diagnosis; HCT, hematopoietic cell transplant; WBC, white blood cell count (K/uL); Plt, platelet count (K/uL); BM, bone marrow; Hb F, fetal hemoglobin; chemo, chemotherapy; GVHD, graft versus host disease; ANC, absolute neutrophil count; Bu, Busulfan; Mel, Melphalan; rATG, rabbit anti-thymocyte globulin; Flu, fludarabine; Alem, alemtuzumab; Ara-C, cytarabine; RA, retinoic acid; HU, hydroxyurea; 6MP, 6-mercatopurine; AML, acute myeloid leukemia; FLAG-Ida, fludarabine, cytarabine, granulocyte colony stimulating factor, Idarubicin; UCB, unrelated cord blood; MMSD, mismatched sibling donor-BM; MSD, matched sibling donor-BM; MUD, matched unrelated donor-BM; MPS, methylprednisolone; MTX, Methotrexate; NR, not reported; Ltd=limited (*resolved with topical steroids).

Patient 6, experienced primary graft failure (PGF) at day +55 and received second (TCR- αβ/CD19-depleted related-haploidentical) transplant with TBI (1200 cGy), flu (160mg/m^2^), thiotepa (10mg/kg), rabbit antithymocyte globulin (3.75mg/kg) and rituximab (200mg/m^2^) and has maintained long-term remission. Patient 15 experienced PGF at day +41 with autologous recovery and remains in complete remission at 18 years follow-up.

At 1-year post-HCT, 92% and 69% of patients with immune-reconstitution data available (n=13) had normal B (CD19+) and T (CD3+) lymphocyte counts, respectively. Pre-HCT performance scores improved in almost all patients post-HCT. Patient 6 has a post-HCT performance score of 80% due to underlying congenital heart defect awaiting surgical repair. At a median follow-up 7.6 (range 2.9-21.5) years, 100% disease-free survival (DFS) and OS were observed. No patients have developed a secondary malignancy or HCT-related organ dysfunction.

## Discussion

JMML is a predominantly aggressive and fatal disorder. In one analysis of patients diagnosed prior to molecular characterization, the probability of survival at 10 years in transplanted patients was 0.39 (standard error [SE] = 0.10) versus 0.06 (SE = 0.04) in non-transplanted patients (6). Prompt HCT is recommended for children with JMML and NF-1, somatic *PTPN-11* and *K-RAS* mutations, and for most children with somatic *N-RAS* mutations (11).

Due to the rarity JMML and paucity of large clinical trials, there is no clear standard conditioning regimen for patients undergoing HCT. One of the largest reports (n=100) used MAC with oral Bu/Mel/cyclophosphamide (Cy), with the goal of avoiding TBI-associated toxicity (3). Transplant related mortality (TRM) was 13% and one-third of patients relapsed (3). Similar TRM/relapse rates were recently reported among patients who received MAC with Bu (oral/IV)/Mel/Flu (12). Therefore, further attempts to reduce transplant related morbidity/mortality without compromising survival have been employed (with conflicting results). The Children’s Oncology Group (COG) conducted a randomized study comparing 2 myeloablative regimens Bu/Flu (n=9) to Bu/Mel/Cy (n=6); however, the trial closed early due to high relapse rates in the Bu/Flu arm (13). In a Japanese registry study (n=129) with a variety of conditioning regimens, 5-year OS, cumulative relapse incidence and TRM were 64%, 34% and 21%, respectively, (with 73% 5-year OS and 26% cumulative relapse incidence in the subgroup (n=59) treated with myeloablative Bu/Flu/Mel) (8).

SOS disproportionately affects infants with a prevalence of 20–60% depending on age and primary disease indication for HCT, compared with 10% in adults (9, 14). In this study, 29% were infants at the time of HCT, and notably, these were the patients who developed SOS. In the COG study, both arms used a single alkylating agent, but SOS developed in 50% of patients on the Bu/Mel/Cy arm and in 22% on the Bu/Flu arm (13). Our results are comparable with Bu/Flu arm of the COG study, suggesting that patients with JMML have an inherently high risk for SOS (15). In 2016, defibrotide was approved for treatment of HCT patients diagnosed with severe SOS. In this study, most cases of SOS were mild, and all cases resolved without defibrotide therapy. Regardless, these collective findings may underscore the potential need for vigilant monitoring/aggressive mitigation strategies for SOS in JMML patients undergoing HCT in the current era. Approximately 35% of children undergoing HCT for various diseases require PICU support in the immediate post-transplant period with PICU mortality of 44% (15). The 100% long-term survival observed in this cohort, suggest that HCT patients with JMML requiring PICU support may have promising outcomes.

Mitigation efforts for PGF included either splenectomy or infusion of IVIG pre-HCT. Splenectomy was historically used for lack of other symptomatic control measures in patients with JMML as it was also associated with decreased transfusion requirements, albeit with increased risk of infection (6). The use of splenectomy to promote engraftment after HCT is no longer routine and typically reserved for the presence of hypersplenism/platelet refractoriness^.6^ In our study, patients received IVIG prior to graft infusion to block the reticuloendothelial system (10) aiming to enhance engraftment. However, the role of IVIG prior to HCT to promote engraftment may warrant prospective investigation. Our improved outcomes with Bu/Mel backbone conditioning regimen are consistent with findings of a recent Japanese study (16), although in this study 6/21 [28.6%] patients (compared to 2/17 [11.8%] patients in our study) had PGF. None of the patients in this Japanese study had splenectomy or IVIG pre-HCT.

The role of GVHD in relapse prevention in JMML is poorly defined. GVHD, particularly chronic GVHD, has been associated with improved survival and lower risk of relapse in JMML post-HCT presumably due to graft-versus-leukemia (GVL) effect (8, 11). However, one of the largest reports did not show benefit of either acute/chronic GVHD (3). The impact of GVHD could not be assessed in this study (100% OS).

While none of the patients in our study received maintenance therapy post-HCT, such therapy may significantly reduce relapse and improve survival in JMML patients. Although FLT3 mutations are not as common in JMML as in acute myeloid leukemia, they can occur and can be a target for maintenance therapy (17). Azacytidine or Decitabine maintenance post-HCT along with donor lymphocyte infusions to enhance GVL effect may salvage patient who have molecular evidence of disease Post-HCT (18, 19). Trametinib maintenance post-HCT for RAS mutated JMML is a promising approach (20).

Majority of patients in this cohort received chemotherapy for cytoreduction prior to HCT resulting in disease debulking, raising the possibility that the high DFS/OS could in part be due to a reduction of the pre-transplant disease burden. At the time of this report, all patients remain alive, disease free, GVHD free, and off all immunosuppression. Evidence of immune reconstitution was observed in most patients by 12 months post-transplant and there was no observed infection- related mortality. Targeted Bu CSS likely contributed to improved outcomes with no TRM, compared with prior reports with oral Bu and/or no TDM.

While conclusive inferences are limited by the small sample size and heterogeneity of the cohort (inherent with the rarity/heterogeneity of this disease), to our knowledge, with 100% OS and DFS, a Bu/Mel backbone conditioning regimen with or without serotherapy or Flu based on stem cell source and donor type, represents the most effective multi-center strategy published to date. This approach warrants prospective investigation.

## Data Availability

The original contributions presented in the study are included in the article/supplementary material. Further inquiries can be directed to the corresponding author.
